# The Evaluation of Gingival Crevicular Fluid Biomarkers as Predictors of Gingival Enlargement in Patients Undergoing Fixed Orthodontic Treatment: A Prospective Study

**DOI:** 10.7759/cureus.74281

**Published:** 2024-11-22

**Authors:** Puja Debnath, Sayeeda Laeque Bangi, Mohammed Feroze Hussain, Shabir Rafiq, Syed Tousifulla, Mufeed Abdu, Seema Gupta

**Affiliations:** 1 Department of Periodontics, Agartala Government Dental College, Agartala, IND; 2 Department of Orthodontics and Dentofacial Orthopedics, Al Badar Rural Dental College and Hospital, Gulbarga, IND; 3 Department of Orthodontics, Medcura Health Care, Bengaluru, IND; 4 Department of Orthodontics and Dentofacial Orthopedics, Kalka Dental College, Meerut, IND; 5 Department of Orthodontics and Dentofacial Orthopedics, Kolar Gold Fields (KGF) College of Dental Sciences and Hospital, Kolar Gold Fields, IND; 6 Department of Orthodontics, Aashraya Dental Clinic, Kozhikode, IND; 7 Department of Orthodontics and Dentofacial Orthopedics, Kothiwal Dental College and Research Centre, Moradabad, IND

**Keywords:** biomarkers, enlargement, fixed, gingival, inflammation, orthodontic, periodontal

## Abstract

Introduction

Gingival enlargement (GE) poses a significant problem during fixed orthodontic treatment (FOT). Thus, the primary aim of the current study was to evaluate the concentrations of biomarkers present in the gingival crevicular fluid (GCF) of individuals receiving FOT. The ancillary aim was to examine and compare biomarker levels among patients exhibiting GE undergoing FOT, those without GE undergoing FOT, and a control group comprising individuals not undergoing FOT and to assess the predictors for GE in patients undergoing orthodontic treatment.

Materials and methods

A cross-sectional observational study was conducted on 129 patients who were divided into three groups: group 1, controls (n=18); group 2, patients who underwent FOT without GE (n=89); and group 3, patients who underwent FOT with GE (n=22). The gingival index (GI), periodontal index (PI), and inflammatory biomarkers such as interleukin 6 (IL-6), transforming growth factor-beta 1 (TGF-β1), tumor necrosis factor-alpha (TNF-α), matrix metalloproteinase 9 (MMP-9), and prostaglandin E2 (PGE2) were estimated by enzyme-linked immunosorbent assay (ELISA) analysis at baseline (T0) and after one year of orthodontic treatment (T1).

Results

GE was observed in 22 (19.81%) of the 111 patients who were undergoing orthodontic treatment. No statistically significant differences were observed between the groups for GI, PI, and biomarkers at T0 (p>0.05), whereas there was a statistically significant increase in the levels of inflammatory biomarkers, GI, and PI in group 3, followed by group 2, compared to group 1 (p<0.05). Group 3 showed the greatest increase in the biomarker levels from T0 to T1 (IL-6, 3.66±1.91 pg/mL; TGF-β1, 7.52±3.85 pg/mL; TNF-α, 16.96±3.82 pg/mL; MMP-9, 30.72±7.07 pg/mL; PGE2, 78.29±20.53 pg/mL). GI and PI were strong predictors of GE, whereas biomarkers were weak predictors.

Conclusion

GE in patients with FOT was significantly associated with an increase in GI and PI due to insufficient oral hygiene, leading to increased levels of inflammatory biomarkers in the GCF.

## Introduction

Orthodontic interventions are frequently conducted as necessary measures to address diverse malocclusions and enhance the aesthetic appearance. This treatment modality is predominantly pursued by adolescents and young adults aged 18-30 years; however, contemporary trends reveal that individuals over the age of 30 years are increasingly seeking orthodontic care primarily for cosmetic purposes due to heightened awareness [1]. The process of orthodontic tooth movement necessitates the exertion of external forces that facilitate the displacement of teeth within their supporting periodontium, thus requiring a healthy periodontal environment to ensure the efficacy of treatment [2]. A notable increase in the volume of the gingiva serves as the defining characteristic of gingival enlargement (GE), which is alternatively referred to as gingival overgrowth. Precisely determining the underlying cause of this enlargement is essential for its effective management, and any enlargement in the anterior portion of the maxilla markedly affects the aesthetic results [3]. The symmetry and form of these gingival components are pivotal in establishing the harmonious aesthetic integrity of both natural and prosthetic dental configurations [4]. In the posterior area, it impedes effective plaque management and exacerbates the condition, leading to the complete encasement of clinical crowns.

The association between fixed orthodontic treatment (FOT) and chronic periodontal conditions warrants further investigation. The accumulation of dental plaque and suboptimal oral hygiene practices contribute to the manifestation of GE. During the course of FOT, patients frequently encounter GE as a consequence of this process; however, the precise underlying mechanisms remain unclear [5,6]. Periodontal tissues must be healthy to ensure effective tooth movement. Nonetheless, when the gingival disease is extensive and self-administered oral hygiene practices are obstructed, nonsurgical periodontal interventions, including scaling and oral hygiene education, prove ineffective and require surgical procedures such as gingivoplasty or gingivectomy [3].

Gingival crevicular fluid (GCF) is a biological fluid that serves both physiological and inflammatory functions derived from the gingival vascular plexus. It is evident that the characteristics of the GCF vary in terms of microbial diversity and the concentration and variety of molecular biomarkers when contrasting healthy sites from diseased subjects with healthy sites from periodontally healthy individuals [7]. Furthermore, there are significant alterations in the GCF composition throughout disease progression, and specific mediators may serve as indicators for forecasting future disease outcomes. For a healthy periodontium, GCF is produced as a result of an osmotic gradient and is very small in quantity, whereas during inflammatory conditions such as GE, it is expressed in greater quantity as an inflammatory exudate and contains biomarkers of inflammation [8]. Thus, the primary aim of the current study was to evaluate the concentrations of biomarkers present in the GCF of individuals receiving FOT. The ancillary aim was to examine and compare biomarker levels among patients exhibiting GE undergoing FOT, those without GE undergoing FOT, and a control group comprising individuals not undergoing FOT and to assess the predictors for GE in patients undergoing orthodontic treatment.

## Materials and methods

Study design and setting

This prospective observational study was conducted in the Department of Orthodontics, Kothiwal Dental College and Research Centre, Moradabad, from January 2022 to May 2024. The study was conducted after obtaining ethical committee approval from the Kothiwal Dental College and Research Centre Institutional Review Board (approval number: KDCRC/IERB/12/2021/82) and followed the principles of the Declaration of Helsinki. Written informed consent was obtained from all patients.

Sample size estimation

To ensure adequate statistical power, the required sample size for this study was estimated based on an 80% confidence level, a 5% margin of error, and a previously reported prevalence of gingival enlargement in orthodontic patients of 65.7% [9]. The sample size calculation used the standard formula for estimating proportions: n=Z2⋅p⋅(1−p)/E2, where n is the required sample size, Z is the Z-score for the desired confidence level (1.28 for 80% confidence), p is the prevalence rate (0.65), and E is the margin of error (0.05). Applying these values, the estimated sample size required was approximately 129 participants, which would provide sufficient power to detect meaningful differences and ensure the reliability of the study's findings.

Participants' eligibility

A total of 580 patients visited the outpatient department (OPD) during the study period. Convenience sampling was adopted. A total of 129 patients were selected for this study based on eligibility criteria. Cases were selected based on the following inclusion criteria: systemically and periodontally healthy patients of any sex, aged 18-30 years, with Angle's Class I mild bimaxillary protrusion, mild Class II Division 1 cases with an overjet of 4-5 mm and end-on molars, crowding not more than 2 mm in the upper arch, requiring the extraction of the upper two premolars, individuals with good oral hygiene with a gingival index (GI) of less than 1 [10], and no clinical attachment loss as diagnosed by periodontal index (PI) [11]. Patients who refused orthodontic treatment were followed up and served as controls. Pregnant and lactating women, smokers, those who had a habit of drinking alcohol, patients with missing teeth, previous history of orthodontic treatment, the presence of gingival disease, a history of periodontal treatment, or the use of corticosteroids or antibiotics in the past year were excluded from the study.

Methodology

**Figure 1:**
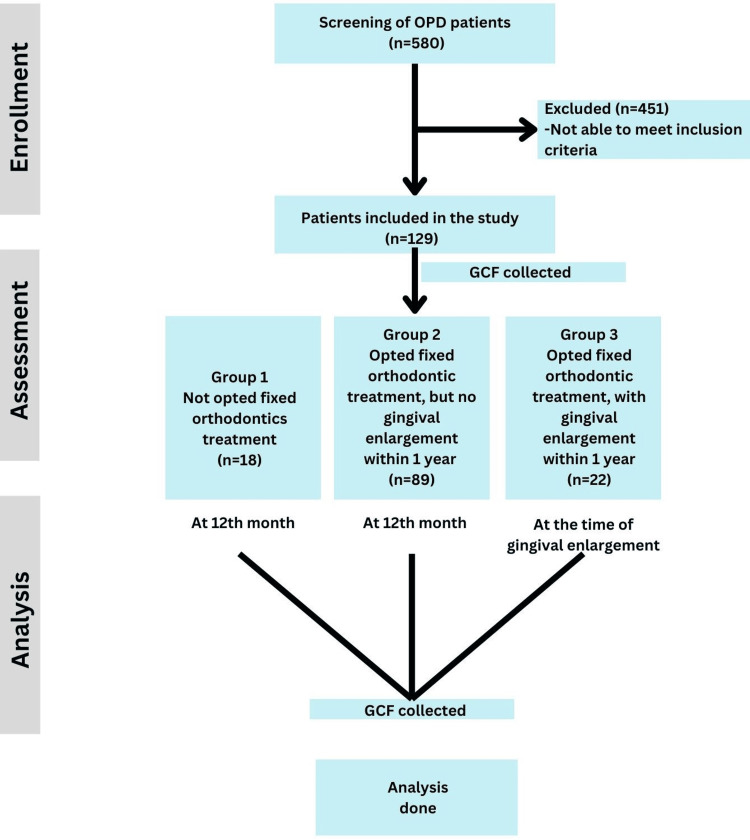
Study design. OPD, outpatient department; GCF, gingival crevicular fluid

Among the 129 participants, 18 patients (13.95%) who declined fixed orthodontic treatment were categorized into group 1 (control). The remaining 111 patients (86.05%) consented to undergo fixed orthodontic treatment. Baseline parameters, including the gingival index (GI), the periodontal index (PI), and other inflammatory biomarkers, were recorded prior to the initiation of treatment (T0). During the follow-up period, spanning 6-12 months, 22 of the 111 patients developed gingival enlargement (GE) and were categorized into group 3, while the remaining 89 patients without GE were assigned to group 2. The flow diagram of the study is shown in Figure 1.

All patients were given similar oral hygiene instructions, such as the use of fluoridated toothpaste twice daily (once in the morning and once at night before going to bed) with a soft-bristled brush and 5 mL of povidone-iodine mouthwash once daily at night.

**Figure 2:**
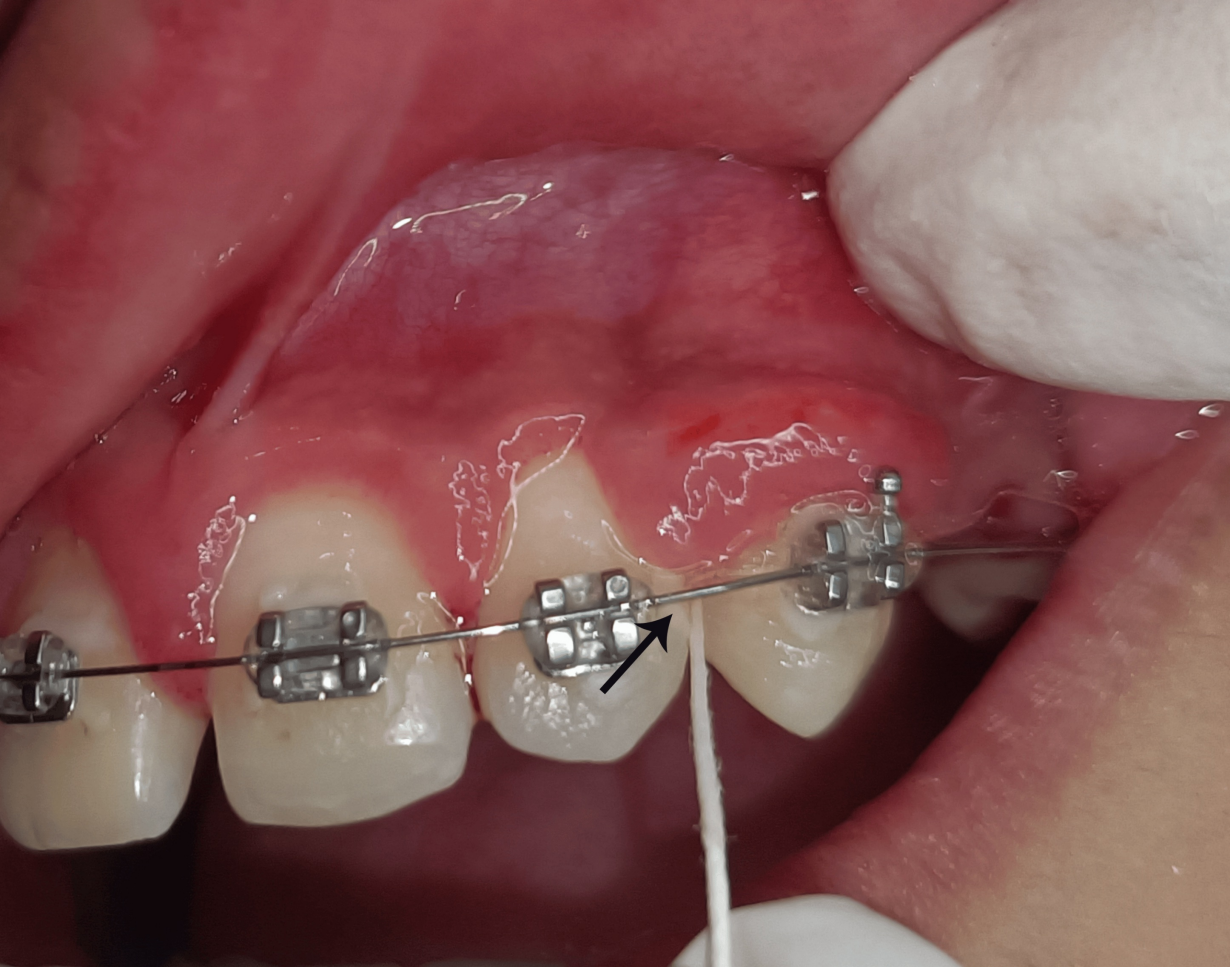
Collection of gingivo-crevicular fluid with paper points from gingival enlargement area at the distal surface of the lateral incisor.

Prior to the designated appointment for the collection of GCF, all participants underwent comprehensive oral prophylactic procedures. The GCF was extracted from the distal sides of all maxillary teeth, specifically from the first premolars on either side (comprising 10 teeth in total), utilizing PerioPaper points (Mani Inc., Utsunomiya, Japan). This methodology presents a plethora of advantages, such as user-friendliness, facilitating the acquisition of samples from specific locations, and a rapid procedure that minimizes tissue damage [12]. The participants were instructed to abstain from eating for a minimum of eight hours preceding the appointment. The designated area was meticulously dried using an air syringe to ensure optimal conditions. The isolation of the site was achieved using cotton rolls or mirror retraction to prevent contamination from blood and saliva. Paper points were carefully inserted into the gingival sulcus of the selected teeth (Figure 2).

After 30 seconds, the paper points were withdrawn, and those that were contaminated with blood were discarded. The remaining samples were placed in a centrifuge tube. Subsequently, 300 μL of phosphate-buffered saline was introduced into the centrifuge tube, which was then incubated overnight at 4°C. The tubes were then centrifuged at 3000×g for five minutes at 4°C. Ultimately, the transparent supernatant was extracted and preserved at -80°C for subsequent enzyme-linked immunosorbent assay (ELISA) analysis to estimate biomarkers such as interleukin 6 (IL-6), transforming growth factor-beta 1 (TGF-β1), tumor necrosis factor-alpha (TNF-α), matrix metalloproteinase 9 (MMP-9), and prostaglandin E2 (PGE2).

Statistical analysis

The data collected were entered into Microsoft Excel (Microsoft Corp., Redmond, WA) for statistical analysis using the SPSS software version 23.0 (for Windows, version 23.0, IBM Corp., Armonk, NY). Normality was assessed using the Shapiro-Wilk test, and the data met normality assumptions. Categorical variables were summarized as frequencies and percentages, and proportional distributions were evaluated using chi-square analysis. Continuous variables are expressed as mean and standard deviation (SD). The analysis of variance (ANOVA) was conducted to compare the means across groups, with post hoc analysis performed using Tukey's test. Intragroup comparisons across time points were assessed using paired t-tests. Additionally, multifactorial logistic regression analysis was used to predict the odds ratio (OR) of gingival enlargement, considering independent factors as predictors.

## Results

**Figure 3:**
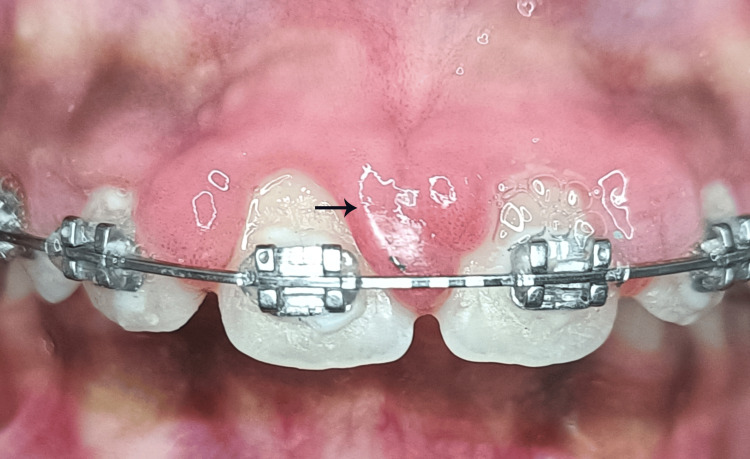
Gingival enlargement at maxillary anterior region.

GE was observed in 22 (19.81%) of the 111 patients who were undergoing orthodontic treatment. Most of these enlargements were noticed in the second premolar region; however, in eight cases, they were also observed in the anterior region (Figure 3).

The comparison of the baseline characteristics of the study groups showed no statistically significant differences between the groups, which suggested that the groups were comparable at baseline, and there was no confounding bias. The distribution of sex showed that group 2 exhibited a greater number of men than group 3, whereas GE was seen more frequently in women. Class II Division 1 malocclusion was a predominant malocclusion in all groups, where the extraction of the upper two premolars was performed as a treatment modality. All groups showed mostly well-aligned arches with minimal crowding of less than 2 mm in four (22.22%) patients in group 1, 31 (34.83%) patients in group 2, and six (27.27%) patients in group 3. The mean age of the patients was 26.5±4.56 years in group 1, 28.5±3.97 years in group 2, and 27.68±3.62 years in group 3 (Table 1).

**Table 1:** Basic characteristics of the study groups. Data such as gender, malocclusions, and crowding are presented in the form of n (%), and age has been presented in the form of mean±standard deviation (SD). P-value>0.05: non-significant. *P-value using chi-square test. **P-value using the analysis of variance (ANOVA) test.

Parameters	Category	Group 1 (n=18, 13.84%)	Group 2 (n=89, 68.46%)	Group 3 (n=22, 16.92%)	Test statistics	P-value
Gender*	Male	9 (50.00%)	47 (42.34%)	8 (36.37%)	1.911	0.384
	Female	9 (50.00%)	42 (37.84%)	14 (63.33%)		
Malocclusion*	Class I bimaxillary protrusion	6 (33.32%)	28 (31.46%)	9 (40.91%)	0.709	0.701
	Class II Division 1	12 (66.67%)	61 (68.54%)	13 (59.09%)		
Crowding*	Yes	4 (22.22%)	31 (34.83%)	6 (27.27%)	1.347	0.509
	No	14 (77.78%)	58 (65.17%)	16 (72.73%)		
Age in years**	Mean±SD	26.5±4.56	28.5±3.97	27.68± 3.62	2.005	0.132

The baseline comparison of GI, PI, and biomarkers showed statistically insignificant differences between the groups, which showed that all groups had similar periodontal conditions at baseline (Table 2).

**Table 2:** Intergroup comparison with the analysis of variance (ANOVA) test at baseline (T0). P-value>0.05: non-significant. Data presented in the form of mean±standard deviation (SD). IL-6, interleukin 6; TGF, transforming growth factor; TNF, tumor necrosis factor; MMP-9, matrix metalloproteinase 9; PGE2, prostaglandin E2

Parameters	Group 1, mean±SD	Group 2, mean±SD	Group 3, mean±SD	F statistics	P-value
Gingival index	0.54±0.49	0.57±0.54	0.59±0.51	0.044	0.952
Periodontal index	0.42±0.53	0.49±0.59	0.67±0.46	1.176	0.316
IL-6 (pg/mL)	1.02±0.58	1.25±0.97	1.60±0.91	2.111	0.125
TGF-beta (pg/mL)	1.25±0.76	1.07±1.03	1.01±1.07	0.731	0.313
TNF-alpha (pg/mL)	1.64±0.85	1.94±1.41	1.78±1.46	0.425	0.654
MMP-9 (pg/mL)	12.25±3.45	13.35±5.34	13.05±6.79	0.314	0.730
PGE2 (pg/mL)	14.56±3.25	15.27±4.54	15.56±8.78	0.185	0.831

The ANOVA test at T1 demonstrated significant intergroup differences, indicating substantial biochemical and inflammatory variations with orthodontic treatment. GE was noticed at 6-12 months after the start of orthodontic treatment in 22 patients. GI and PI showed marked differences among all groups (p=0.001), with Tukey's post hoc analysis revealing significant differences between each pair of groups. Group 3 had the highest PI and GI scores, indicating marked inflammation. All biomarkers were significantly higher in group 3, followed by group 2, than in the control group, indicating higher inflammatory markers with orthodontic treatment. It also suggests that GE is associated with high levels of inflammatory biomarkers in GCF, as shown in Table 3.

**Table 3:** Intergroup comparison at T1 time stamp with ANOVA test, followed by post hoc Tukey's test. Data presented in the form of mean±standard deviation (SD). *P-value<0.05: significant. IL-6, interleukin 6; TGF, transforming growth factor; TNF, tumor necrosis factor; MMP-9, matrix metalloproteinase 9; PGE2, prostaglandin E2; G1, group 1; G2, group 2; G3, group 3; ANOVA, analysis of variance

Parameters	Group 1, mean±SD	Group 2, mean±SD	Group 3, mean±SD	F statistics	P-value	Post hoc analysis
Gingival index	0.64±0.42	1.79±0.65	2.36±0.49	42.40	0.001*	G1 versus G2, G1 versus G3, and G2 versus G3
Periodontal index	0.72±0.56	1.43±0.67	2.55±0.51	44.56	0.001*	G1 versus G2, G1 versus G3, and G2 versus G3
IL-6 (pg/mL)	1.18±0.52	2.23±1.08	5.26±2.81	47.09	0.001*	G1 versus G2, G1 versus G3, and G2 versus G3
TGF-beta (pg/mL)	1.28±0.85	4.70±2.61	8.53±4.88	29.98	0.001*	G1 versus G2, G1 versus G3, and G2 versus G3
TNF-alpha (pg/mL)	1.98±0.81	5.74±2.06	18.74±5.28	233.99	0.001*	G1 versus G2, G1 versus G3, and G2 versus G3
MMP-9 (pg/mL)	10.25±3.75	18.25±5.28	43.77±13.83	132.67	0.001*	G1 versus G2, G1 versus G3, and G2 versus G3
PGE2 (pg/mL)	12.56±4.15	33.97±4.09	93.85±29.21	257.34	0.001*	G1 versus G2, G1 versus G3, and G2 versus G3

Intragroup comparative evaluation using a paired t-test to assess parameters between T0 and T1 revealed significant discrepancies among the groups, particularly within groups 2 and 3. Group 1, designated as the control group, did not demonstrate statistically significant alterations in any biomarker level, GI, or PI. Conversely, substantial increases were noted in both groups 2 and 3 concerning GI, PI, and all evaluated biomarkers, underscoring the marked inflammatory and biochemical responses in these cohorts. This augmentation was more pronounced in group 3 than in group 2. This indicates that orthodontic interventions affect the oral hygiene of patients, resulting in a deterioration of periodontal health, which, in conjunction with the inflammatory responses linked to tooth movement, culminates in elevated levels of inflammatory biomarkers, which, if exceeding a specific threshold, may lead to GE (Table 4).

**Table 4:** Intragroup comparison with paired t-test between T0 and T1. *P-value<0.05: significant. IL-6, interleukin 6; TGF, transforming growth factor; TNF, tumor necrosis factor; MMP-9, matrix metalloproteinase 9; PGE2, prostaglandin E2

Parameters	Group 1 (n=18)	Group 2 (n=89)	Group 3 (n=22)
Gingival index	0.515	0.001*	0.001*
Periodontal index	0.108	0.001*	0.001*
IL-6 (pg/mL)	0.389	0.004*	0.001*
TGF-beta 1 (pg/mL)	0.911	0.001*	0.001*
TNF-alpha (pg/mL)	0.226	0.001*	0.001*
MMP-9 (pg/mL)	0.105	0.217	0.001*
PGE2 (pg/mL)	0.116	0.007*	0.001*

Multivariate logistic regression analysis delineated notable predictors for the manifestation of GE. The OR for GI was 4.46, indicating a robust correlation with gingival enlargement. PI exhibited a considerable OR of 3.28, thereby reinforcing its role. In contrast, all biomarkers demonstrated weak correlations, demonstrating their role as weak predictors of GE. Sex (female), age, and malocclusion were not significant predictors of GE (Table 5).

**Table 5:** Multivariate logistic regression analysis for the occurrence of gingival enlargement. *P-value<0.05: significant. IL-6, interleukin 6; TGF, transforming growth factor; TNF, tumor necrosis factor; MMP-9, matrix metalloproteinase 9; PGE2, prostaglandin E2; CI, confidence interval; LL, lower limit; UL, upper limit

Variables	Standard error	P-value	Odds ratio	95% CI (LL-UL)
Age	0.06	0.178	1.09	0.96-1.23
Gender (female)	0.5	0.214	1.85	0.7-4.9
Gingival index	0.43	0.001*	4.46	1.91-10.42
Periodontal index	0.81	0.001*	3.28	1.23-8.76
Malocclusion (Class II Division 1)	0.51	0.426	0.67	0.25-1.81
TGF-beta 1 (pg/mL)	0.09	0.001*	0.72	0.61-0.85
IL-6 (pg/mL)	0.2	0.001*	0.4	0.27-0.6
TNF-alpha (pg/mL)	0.12	0.001*	0.6	0.48-0.76
MMP-9 (pg/mL)	0.07	0.001*	0.75	0.65-0.86
PGE2 (pg/mL)	0.04	0.001*	0.88	0.82-0.95

## Discussion

The results of the present study indicated that orthodontic treatment leads to deterioration in the periodontal health of the patient, leading to an increase in GI and PI over a period of one year of orthodontic treatment. This finding is supported by previous studies [13-15]. FOT may facilitate the development of supragingival biofilms and exacerbate the state of periodontal tissues. As dental biofilms serve as a significant etiological contributor to gingivitis, inadequate oral hygiene practices can elevate the likelihood of periodontal inflammation. Despite the need for orthodontic patients to receive comprehensive guidance on effective oral hygiene from the outset of their treatment, sustaining adequate oral hygiene can often present considerable challenges for these individuals, and the application of orthodontic forces can induce inflammatory responses within the periodontium. This inflammatory reaction is crucial for the process of orthodontic tooth movement [15].

It has been further revealed that in a few patients undergoing FOT, GE developed and showed increased levels of inflammatory biomarkers in GCF, with an increase in GI and PI. Shirbhate et al. [4], Gong et al. [5], and Rauten et al. [16] also reported this finding. Surlin et al. observed increased levels of MMP-8 in the first few hours of patients undergoing FOT, whereas in cases of GE, the levels of MMP-9 were found to be significantly higher than those in patients who did not develop GE [17]. MMP-9 is also expressed in healthy controls, demonstrating its physiological role in regeneration and remodeling. Similar results have been reported by Orozco-Páez et al. [9].

Prostaglandins, which are biologically active lipid compounds synthesized through the metabolic pathways involving arachidonic acid, are consistently present in elevated concentrations at various loci characterized by inflammatory processes. In recent scientific investigations, the concentrations of PGE2 present within the GCF have been documented to exhibit a positive correlation with the severity of periodontal inflammation, as well as the likelihood of subsequent tissue destruction [18]. This relationship underscores the critical role that PGE2 plays in the pathophysiology of periodontal diseases and highlights its potential as a biomarker for assessing the extent to which inflammatory conditions affect periodontal tissues.

TNF-α is a critical proinflammatory cytokine implicated in osteoclastogenesis. In particular, this cytokine is instrumental in the initiation of osteogenesis, maturation of osteoclasts, and sustenance of osteoclast functions within alveolar bone regions subject to resorption, thereby facilitating tooth movement [14]. Previous studies have reported an increase in TNF-α levels in patients undergoing FOT compared to controls [19,20]. Previous studies have also reported increased levels of IL-6 during FOT, suggesting its role in bone resorption [21,22]. Due to insufficient oral hygiene in the presence of FOT, inflammatory biomarkers, such as TNF-α and IL-6, which is the most potent interleukin, are elevated. This increase was more pronounced in cases of FOT with GE [23].

Our study further indicated that GI and PI are strong predictors of GE compared to inflammatory biomarkers, which are weak predictors. This finding was supported by previous studies, where significant GE was observed in patients undergoing FOT, which could be due to difficulty in maintaining proper oral hygiene, leading to inflammation and the increased release of inflammatory biomarkers, further worsening the situation, and promoting bone resorption [4-6]. Furthermore, GE was seen more in women, which could have been due to the effect of sex hormones, such as estrogen and progesterone [24].

Clinical implications of the study

It is imperative to propose that the meticulous management and control of periodontal health should be systematically and rigorously conducted not only prior to the initiation of orthodontic treatment but also continuously throughout the duration of the treatment process, as well as following its completion, which emphasizes the need for ongoing motivation and comprehensive education regarding proper oral hygiene practices, particularly after the placement of orthodontic appliances, in addition to implementing regular monitoring and evaluations during the entire course of orthodontic care.

Limitations of the study

Because of the cross-sectional study design, it was not feasible to determine the causal relationships between various factors. Furthermore, we did not conduct a microbial analysis to determine the exact reason for the GE found in our patients. However, the quantification of dental plaque was not conducted. The present study was conducted only in the maxillary arch due to financial constraints. Therefore, long-term, prospective studies are required. Another limitation was disproportionate samples in three groups, as small sample sizes in one or more groups reduce the power to detect significant effects within those groups, making it harder to draw conclusions about their behavior or outcomes.

## Conclusions

Based on the findings of the present study, it was concluded that FOT increases the levels of inflammatory biomarkers in GCF, with an increase in GI and PI. Furthermore, these levels drastically increased in cases of GE with FOT. GI and PI were strong predictors, whereas biomarkers such as TNF-α, MMP-9, IL-6, TGF-beta 1, and PGE2 were weak predictors of GE. GE was noticed more frequently in women in 20% of cases with FOT. Gender (female), age, and malocclusion were not statistically significant predictors of GE.
